# Modeling amplified p53 responses under DNA-PK inhibition in DNA damage response

**DOI:** 10.18632/oncotarget.15062

**Published:** 2017-02-03

**Authors:** Tingzhe Sun, Xinda Li, Pingping Shen

**Affiliations:** ^1^ School of Life Sciences, AnQing Normal University, AnQing, Anhui, 246011, China; ^2^ State Key Laboratory of Pharmaceutical Biotechnology and MOE Key Laboratory of Model Animal for Disease Study, Model Animal Research Center, Nanjing University, Nanjing, 210023, China

**Keywords:** amplified p53 pulse, DNA-PK inhibitor, cell fate, mutual information, robustness

## Abstract

During DNA double strand breaks (DSBs) repair, coordinated activation of phosphatidylinositol 3-kinase (PI3K)-like kinases can activate p53 signaling pathway. Recent findings have identified novel interplays among these kinases demonstrating amplified first p53 pulses under DNA-PK inhibition. However, no theoretical model has been developed to characterize such dynamics. In current work, we modeled the prolonged p53 pulses with DNA-PK inhibitor. We could identify a dose-dependent increase in the first pulse amplitude and width. Meanwhile, weakened DNA-PK mediated ATM inhibition was insufficient to reproduce such dynamic behavior. Moreover, the information flow was shifted predominantly to the first pulse under DNA-PK inhibition. Furthermore, the amplified p53 responses were relatively robust. Taken together, our model can faithfully replicate amplified p53 responses under DNA-PK inhibition and provide insights into cell fate decision by manipulating p53 dynamics.

## INTRODUCTION

Faithful repair of DNA damage is critically important for genomic integrity. Cells have evolved multiple strategies to cope with DNA damage by inducing cell cycle arrest, senescence or apoptosis [1]. One of the most detrimental DNA damages is DNA double strand breaks (DSBs). Defect in DNA damage response (DDR) may favor a tumor-prone phenotype [2]. Therefore, the link between dysfunctional DDR and tumor development defines the importance of DNA damage repair.

Sensing DNA double strand breaks is facilitated by phosphatidylinositol 3-kinase (PI3K)-like kinase (PIKK) family members including ATM (ataxia telangiectasia-mutated), ATR (ataxia telangiectasia and Rad3-related protein), and DNA-PK (DNA-dependent protein kinase). The kinetic modifications of PIKK members may dictate DSB repair pathway choice [3]. ATM primarily responds to DNA double strand breaks while recent findings have implied that ATR is also involved in DSB repair by activating end processing [4]. Instead, DNA-PK contributes largely to a DSB repair process called nonhomologous end joining (NHEJ) [1]. The activation of PIKK kinases may relay signals to tumor suppressor p53 [5].

The dynamics of p53 in DDR has long been an active area of research. The p53 can induce the expression of its negative regulator MDM2, which targets p53 for degradation [6]. A second negative loop involves p53 mediated Wip1 induction, which in turn deactivates upstream PI3K-like kinases to terminate p53 activation [7]. These two negative feedback loops impel pulsatile p53 dynamics [8]. Alterations in uniform p53 pulses may dictate cell fate [9, 10]. Previous studies at single cell level showed uniform p53 pulses under γ-irradiation [11, 12]. More dynamical p53 patterns have been identified depending on the radiation type and dose [10, 13, 14]. A most recent finding by Finzel et al. has demonstrated a novel p53 dynamics in response to DNA damage [15]. Their study argues that ATR or DNA-PK alone could compensate for the deficiency in the other two PI3K-like members and retain regular p53 dynamics. Instead, in the absence of DNA-PK, p53 reacts more strongly to ATM mediated phosphorylation with escalated first pulses [15]. ATM is hyper-activated when catalytic DNA-PK activity is blocked, implying that DNA-PK may inhibit ATM kinase activity [15, 16].

Previous mathematical models have not explored the prolonged activation of the first p53 pulse under DNA-PK inhibition [11, 12, 15, 17, 18]. Therefore, we constructed a simplified mathematical model which incorporated novel interplay among PIKK family members as well as their coordinated activation of downstream effector p53 module. We found that p53 showed enlarged first pulses with increased amplitude and duration when DNA-PK was inhibited. The enhanced activation may depend on irradiation dose. A pair-wise inhibition of PIKK members also confirmed that the p53 reacted strongly to DNA damage in cells with functional ATM and in the absence of DNA-PK irrespective of ATR. We conjectured that the DNA-PK mediated inhibition of ATM might be moderate. We also identified that the mutual information displayed a first pulse predominance with DNA-PK inhibition. The amplified p53 pulses by inhibiting DNA-PK were relatively robust to fluctuating parameters. Our study characterized the interplay among PIKK members and p53 while the amplified p53 response might provide clues to cell fate decision.

## RESULTS

### Amplified p53 responses under DNA-PK inhibition

**Figure 1:**
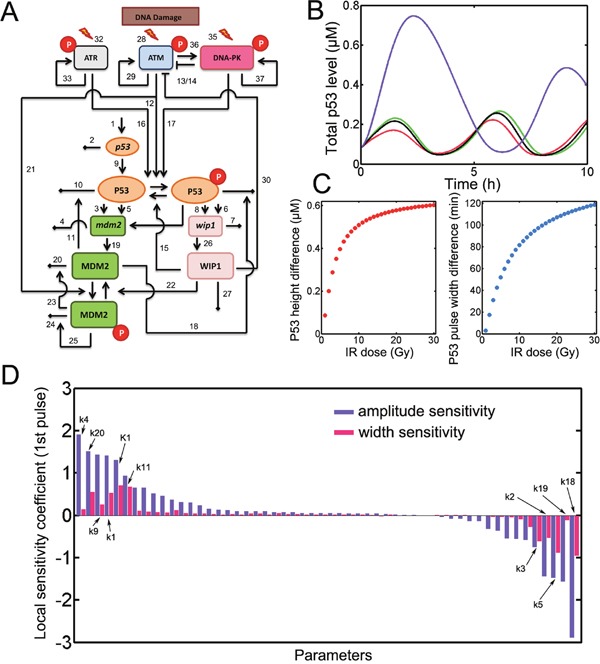
model construction **A**. the schematic diagram for p53 model. Numbers denote the parameters listed in Supplementary Table 2. Notably, the numbers corresponding to basal deactivation rate of activated ATM, ATR and DNA-PK (31, 34 and 38) were not shown in the diagram. Red circle represents phosphorylation. **B**. Deterministic simulation of the model for wild type (WT, green), ATM inhibition (red), ATR inhibition (black) and DNA-PK inhibition (violet). IR = 10 Gy. **C**. The difference of the amplitude and full-width at half-maximum (FWHM) for first p53 pulses under DNA-PK and WT conditions. **D**. Local sensitivity coefficient for the amplitude and FWHM of the first p53 pulse. Representative sensitive parameters were marked with numbers.

We constructed the model incorporating two negative feedback loops in p53 signaling and kinetic interplay among PIKK members (Figure 1A, for details, see supplemental methods). We found that p53 accumulated in sustained pulses in response to a 10 Gy radiation (Figure 1B). As Finzel et al. have focused on exploring the first p53 pulse after irradiation [15], we only quantified the first p53. Applying ATM or ATR inhibitors did not substantially influence the shape of the pulses (Figure 1B). However, after DNA-PK inhibition, we found that the first p53 pulse was substantially amplified in both duration and amplitude (Figure 1B). The increments in amplitude and duration also depended on the irradiation doses (Figure 1C). Sensitivity analysis identified that MDM2 turnover (*k_4_* and *k_20_*) and p53 production rates (*k_9_* and *k_1_*) were among the most positive parameters for p53 amplification (Figure 1D and Supplementary Table 2). Accordingly, increased phosphorylated p53 degradation (*k_18_*), *mdm2* production (*k_19_ k_3_* and *k_5_*) and *p53* degradation (*k_2_*) substantially suppressed p53 pulses (Figure 1D and Supplementary Table 2). We also noticed that the overall amplitude sensitivities exceeded width sensitivities (Figure 1D) implying that the width of the pulses was relatively robust to parametric variation [11]. To further investigate p53 dynamics, we turned to stochastic simulations.

**Figure 2:**
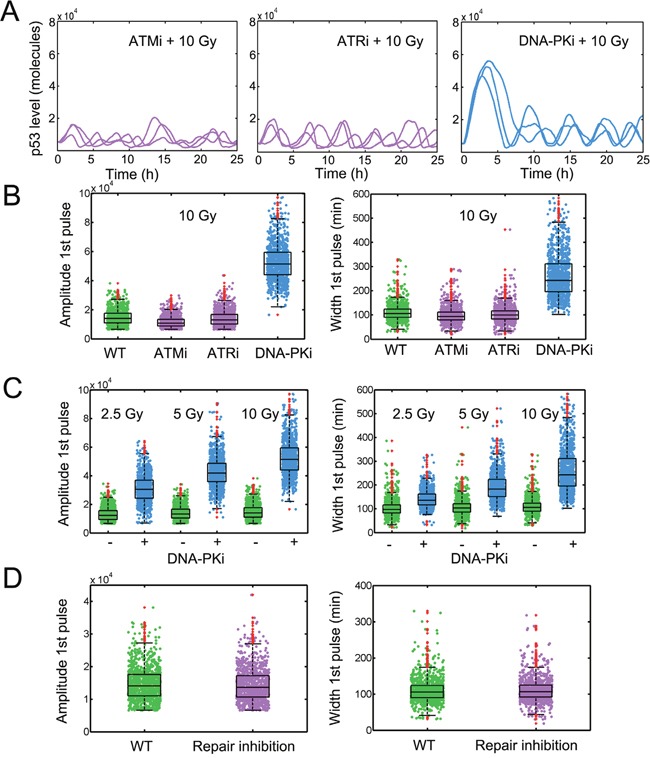
Amplified p53 pulses under DNA-PK inhibition **A**. Stochastic simulation of temporal p53 series under ATM inhibition (ATMi, left), ATR inhibition (ATRi, middle) and DNA-PK inhibition (DNA-PKi, right). IR = 10 Gy. **B**. Calculation of the amplitude (left) and full-width at half-maximum (FWHM, right) of the first p53 pulse under wild type (WT), ATMi, ATRi and DNA-PKi conditions. IR = 10 Gy. 1000 simulations were shown. **C**. Characteristics of first p53 pulses in cells either left untreated or treated with DNA-PKi in response to a 2.5 Gy, 5 Gy and 10 Gy irradiation. **D**. Quantifying the amplitude and FWHM of first p53 pulses under WT and repair inhibition conditions (*k_fix1_*’= 0.01). In boxplots, the red dots denote outliers.

P53 displayed consecutive pulses and DSBs were gradually repaired under a 10 Gy irradiation (Supplementary Figure 2A and 2B). When all PIKK inhibitors were added, p53 only fluctuated at low levels and showed no obvious pulses consistent with Finzel et al’s results [15] (Supplementary Figure 2A). When we only applied ATM inhibitor, we did not observe substantial differences in p53 pulses compared with untreated control (Figure 2A, left and Supplementary Figure 2). We only observed small reduction in first p53 pulses (Figure 2B, Supplementary Figure 3A and 3B). We further used ATR inhibitor and found qualitatively similar results (Figure 2A, middle, Figure 2B, Supplementary Figure 3A and 3B). However, when we finally inhibited DNA-PK activities, the first p53 pulses were strongly enlarged in both amplitude and duration (Figure 2A, right, Figure 2B, Supplementary Figure 3A and 3B).

We next investigated whether the amplified p53 responses correlated with the irradiation dose as previously described [15] by quantifying the p53 first pulses with or without DNA-PK inhibitor at different irradiation doses. The results showed that the characteristics of first p53 pulses were hardly changed with different doses (Figure 2C, green boxes, Supplementary Figure 3C and 3D). However, once the DNA-PK inhibitor was applied, we found a dose-dependent increase in both amplitude and duration of p53 pulses (Figure 2C, blue boxes, Supplementary Figure 3C and 3D). Meanwhile, the dynamical patterns of first p53 pulses are insensitive to changes in repair rates (set *k_fix1’_* = 0.01, Figure 2D), implying that increases in DSBs alone are insufficient to induce an amplified p53 responses [15]. Taken together, these results suggested that p53 may undergo enhanced first pulses with DNA-PK inhibition in a dose dependent manner.

### Enhanced activation of ATM by loss of DNA-PK alters p53 responses

**Figure 3:**
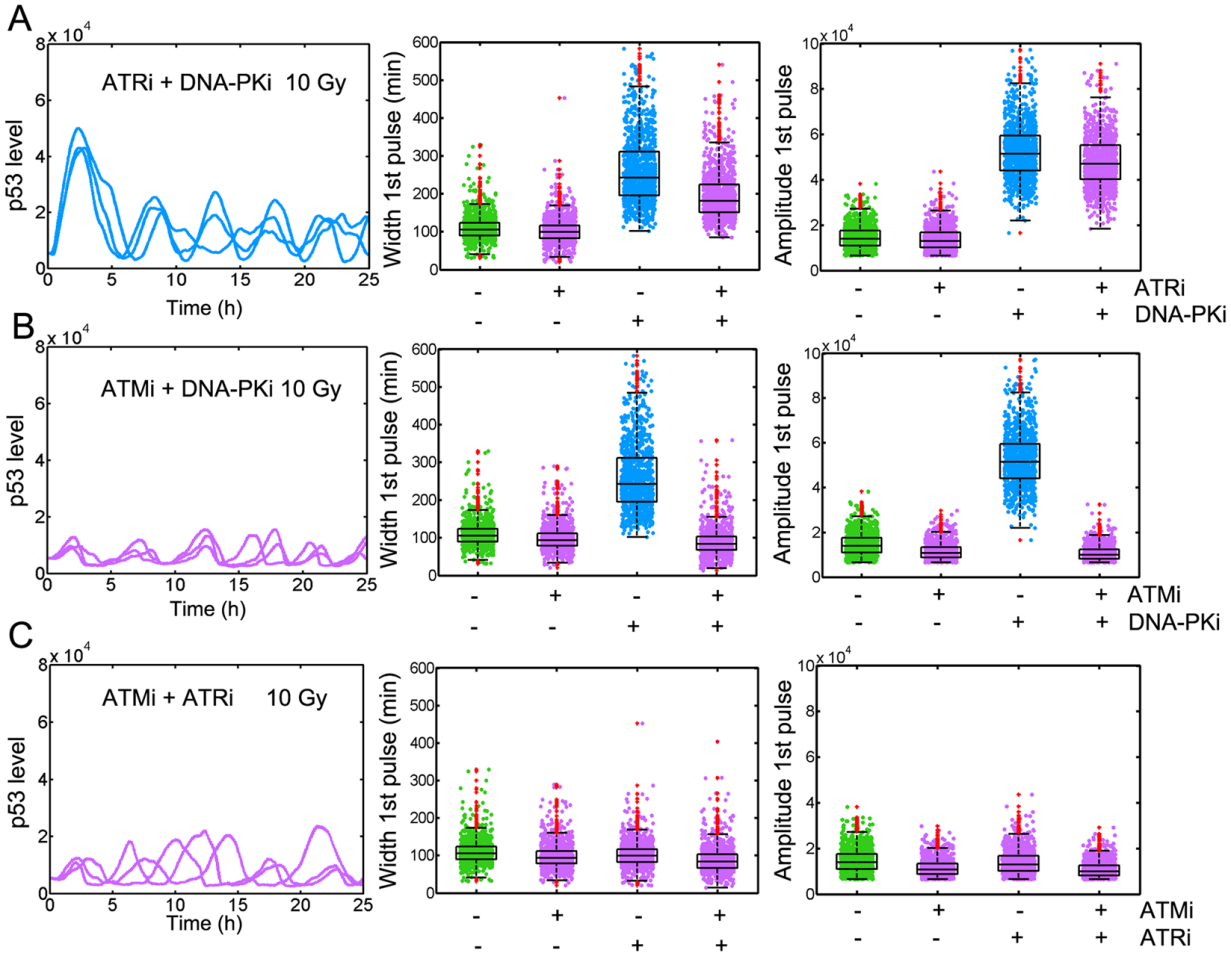
ATM hyper-activation with DNA-PK inhibition contributes to elevated p53 responses Temporal trajectories of p53 (left) and measurement of FWHM (middle) and amplitude (right) in ‘cells’ untreated or treated with **A**. ATRi and DNA-PKi, **B**. ATMi and DNA-PKi, **C**. ATMi and ATRi in combinatorial manner or alone. In boxplots, the red dots denote outliers. Three representative time series were shown.

We next checked whether inhibition of DNA-PK altered the regulatory patterns among PIKK members. We performed pair-wise *in silico* inhibition as described in experiments and reported by Finzel *et al*. [15]. We found that simultaneous inhibition of ATR and DNA-PK restored the amplified p53 accumulation compared with that under wild type condition (Figure 3A and Supplementary Figure 4A). However, combined ATM and DNA-PK inhibition failed to effectively activate p53 (Figure 3B and Supplementary Figure 4B). The width of first p53 pulse was elevated over 200 min in groups treated with DNA-PK inhibitor alone. However, the amplified p53 pulse was diminished when further applying ATM inhibitor (Figure 3B and Supplementary Figure 4B). The amplitude was similarly changed. When ATM and ATR were inhibited, p53 dynamics had no markedly changes (Figure 3C and Supplementary Figure 4C). We further quantified the levels of total activated ATM, p53 and MDM2 post damage with or without DNA-PK inhibitor. We found that total p53 was significantly upregulated under DNA-PK inhibition (Supplementary Figure 5A). Accordingly, MDM2 was severely decreased (Supplementary Figure 5B). Furthermore, activated ATM was also increased after DNA-PK inhibition (Supplementary Figure 5C). These results are consistent with Finzel et al.’s experiments and suggested that exaggerated p53 responses are largely ascribed to hyper-activation of ATM in the absence of DNA-PK independent of ATR status.

### Weak inhibition of ATM by DNA-PK is insufficient to fully reproduce p53 dynamics

**Figure 4:**
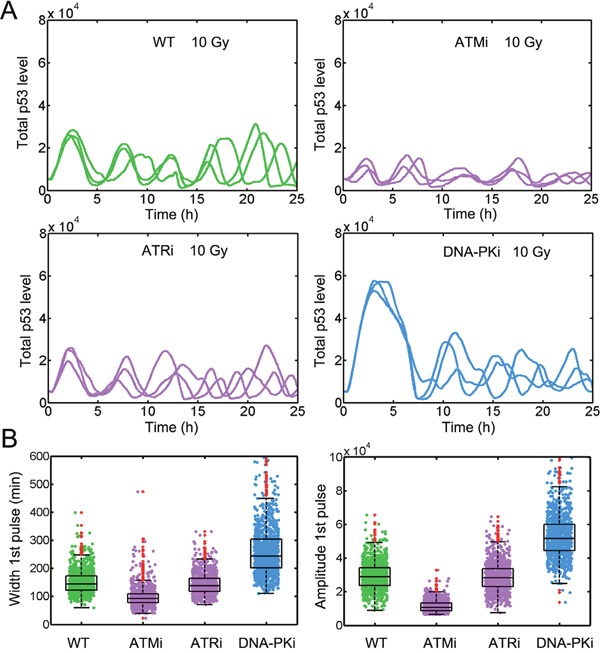
Moderate inhibition of ATM by DNA-PK is required to replicate the p53 dynamics **A**. Time series of p53 under WT (top left), ATMi (top right), ATRi (bottom left) and DNA-PKi (bottom right) conditions when DNA-PK mediated ATM inhibition is relatively weak (*k_13_* = 0.5). Three representative p53 trajectories were shown. IR = 10 Gy. **B**. Quantifying FWHM (left) and amplitude (right) for the first p53 pulses under indicated conditions as described in (A). The red dots denote outliers.

We then explored the required strength of DNA-PK mediated ATM inhibition. We modified the parameter which denotes DNA-PK induced ATM inhibition (*k_13_*= 0.5, 1000 sets, see Supplementary Table 1 and 2). Under wild type conditions, the p53 performed regular pulses similar to those under stronger inhibition condition (Figure 4A, 4B and Supplementary Figure 2). Consistently, DNA-PK inhibitor treatment also induced prolonged p53 activation (Figure 4A and 4B). However, once ATM was inhibited, the FWHM and amplitude of first p53 pulses were severely decreased contrary to the slight reduction in experiments (Figure 4B) [15]. These results suggested at least a moderate inhibition of ATM by DNA-PK is required to replicate the experiments.

### Shifted information propagation under DNA-PK inhibition

**Figure 5:**
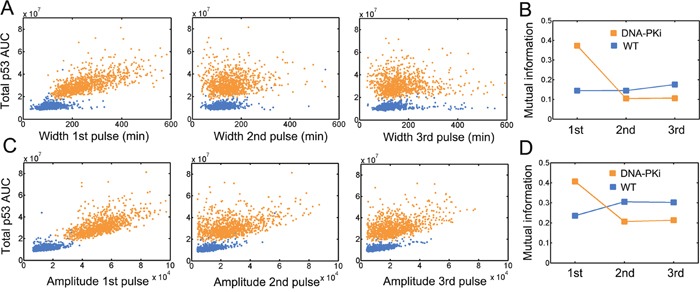
Changes in information flow under DNA-PK inhibition Quantification of the area under curve (AUC) under wild type (blue) and DNA-PK inhibition (orange) conditions. The association of the indicated p53 pulse width **A**. or amplitude **C**. with total p53 AUC were shown in scatter plots. 1000 simulations were run for each condition (i.e. wild type and DNA-PKi). Notably, points at middle and right panels were less than 2000 due to the fact that a fraction of simulations only showed one or two pulses. The integral was evaluated up to 25 hours. Mutual information for indicated pulse width **B**. and amplitude **D**. under wild type (blue) and DNA-PK inhibition (orange) conditions.

We next investigated how information processing was shaped with DNA-PK inhibition. We evaluated the characteristics of consecutive pulses from stochastic p53 trajectories and then explored their correlation with temporal p53 integral. We found that upon DNA-PK inhibition, the temporal accumulation of total p53 was significantly upregulated (Figure 5A and 5C). There were strong signs of correlation between widths of the first pulses and p53 accumulation after DNA-PK inhibition (Figure 5A). The correlation was lowered for subsequent pulses (Figure 5A). The information was near uniformly encoded for consecutive p53 pulses under wild type condition (Figure 5B, blue). Once DNA-PK inhibitor was applied, the information flow was shifted towards the first p53 pulses (Figure 5B, orange). The information flow in subsequent pulses was also decreased compared with that under wild type condition (Figure 5B). Similar tendency was also observed for the amplitude of p53 pulses (Figure 5C and 5D). These results suggested that amplified p53 responses following DNA-PK addition may shift the information flow towards the first pulse.

### Quantifying robustness in p53 dynamics under DNA-PK inhibition

**Figure 6:**
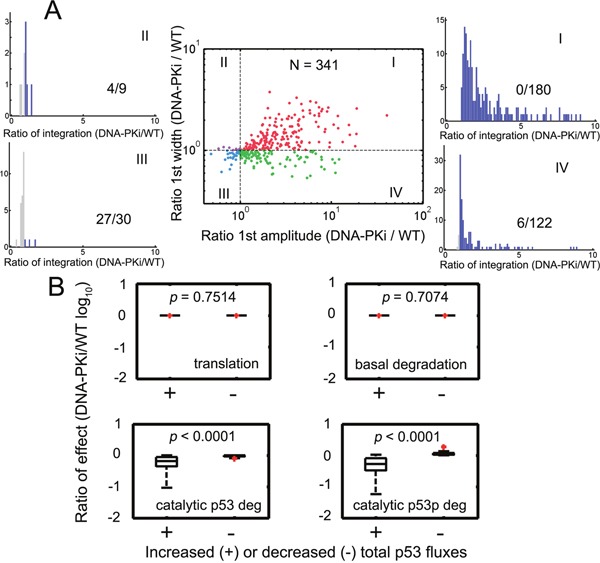
Stochastic parameters identified robust p53 amplification under DNA-PK inhibition **A**. All kinetic parameters were varied by 2-fold with respect to their reference values simultaneously and then the first p53 pulses were compared under wild type and DNA-PK inhibition conditions. 1000 simulations were run and those parameter sets (341 out of 1000) leading to sustained pulses under both conditions were displayed. Horizontal and vertical guidelines were presented as dashed lines. Ratios of the first pulse width and amplitude under DNA-PK inhibition (DNA-PKi) and wild type (WT) conditions were plotted. The histograms denote the distribution of the ratios of total p53 integrals under DNA-PKi and WT conditions. Ratios > 1 were colored blue and those <1 were colored gray. The (number of points <1)/(total points) in each quadrant was shown. The data in I, II, III and IV quadrants were represented as red, violet, cyan and green points, respectively. **B**. Quantification of the ratio of the normalized effects (*k_9_*:*p53* translation; *k_10_*: basal p53 degradation; *k_11_*’·[MDM2]/([P53]+*K_3_*): the E3-ligase MDM2 induced p53 degradation; *k_18_*’·[MDM2]/([P53p]+*K_8_*): MDM2 catalyzed p53p degradation; i.e. the reaction rates divided by corresponding species) under DNA-PKi and WT conditions for increased or decreased total p53 fluxes in (A). Totally, 0 (I) + 4 (II) + 27 (III) + 6 (IV) cases were decreased in total p53 integral. The remaining points were raised in total p53 integrals. The *p* values were shown in each panel. The Mann-Whitney test was used. The red points denote outliers.

We further evaluated how parametric variations may affect the p53 dynamics. We simultaneously varied all kinetic parameters by 2-fold and then investigated whether p53 responses were preserved. 1000 random parameter sets were generated. We found that 341 parameter sets can retain regular p53 under both wild type and DNA-PK inhibition conditions. Furthermore, systems with 180 out of 341 sets (52.79 %) were amplified for both amplitude and width of the first p53 pulses (Figure 6A, red). However, there were only 30 parameter sets leading to reduced amplitude and pulse width (Figure 6A, blue). The remaining ones can result in either amplified width or amplitude (Figure 6A, violet and green). Since p53 can function as a transcription factor and dictates downstream effector expression [19], we further evaluated how the integrated p53 responses were influenced by parametric stochasticity. Simulation showed that the temporal p53 integral was increased when both the amplitude and width of the first pulses were amplified (Figure 6A, the distribution for the 1^st^ quadrant). For those where only the amplitude was enlarged, we also found elevated p53 levels in over 95 % (116/122) cases under DNA-PK inhibition (Figure 6A). However, once the amplitude was reduced, the integrated p53 responses were attenuated with higher probability (Figure 6A). Overall, the accumulated p53 responses under DNA-PK inhibitor treatment can be amplified generally in 89.15 % (304/341) cases. We then measured the flux ratios that directly affected total p53 levels (Supplementary Table 1, the fluxes were normalized by corresponding species). In rare cases where total p53 integral was lowered (37/341), the catalytic degradation for different p53 species was significantly upregulated while basal degradation and the translation remained unaltered (Figure 6B, Mann-Whitney test). These results suggested that the generalized p53 amplification with DNA-PK inhibitor treatment can be preserved provided that the system can perform regular p53 pulses unless MDM2 catalyzed p53 degradation was substantially augmented.

## DISCUSSION

Our results showed that dynamic p53 responses were amplified under DNA-PK inhibition. The amplification of p53 pulses was dose-dependent. Meanwhile, prolonged p53 pulses can give rise to altered cell fate as exemplified by integrated p53 responses (i.e. the integral of temporal p53 series within 25 hours). The local sensitivities for amplitude are larger in absolute values than those for pulse width (Figure 1D) which is consistent with earlier findings [11].

The PIKK family members can all phosphorylate and activate p53 [20]. However, ATM can further activate MDM2 at Ser395 allowing MDM2 self-degradation [21]. Therefore, enforced self-degradation of MDM2 as well as p53 stabilization results in prolonged p53 pulses compared with the effect of ATR or DNA-PK alone.

The kinetic interplay between ATM and DNA-PK can either be mediated by direct inhibition or indirect inhibition via functional deficiency of DNA-PK at break sites [15]. Inactive DNA-PK has been shown to inhibit DSB processing and decrease the repair rates [22]. Therefore, accumulated unrepaired DSBs under DNA-PK inhibition may continuously signal to ATM and activate p53. To verify whether indirect ATM hyper-activation under DNA-PK inhibition can replicate p53 dynamics, we removed the inhibitory effect (*k_13_* = 0) and integrated the system with increased initial DSBs (i.e. 50 DSBs). However, we failed to detect a dose-dependent increase in pulse amplitude or width (Supplementary Figure 6). In Finzel et al.’s experiments, the PIKK inhibitors were added only 30 minutes before irradiation [15], during which the intrinsic DSBs might not even be accumulated in sufficient amount. Therefore, indirect effect may not amplify p53 pulses. During the review, Zhou et al. identified that DNA-PK phosphorylates ATM at multiple sites and inhibits ATM activity [23]. Our model inference is consistent with Zhou et al.’s study.

We further identified that DNA-PK inhibition may reinforce the information flows to the first p53 pulses (Figure 5). Among the numerous types of DNA damage, DNA double strand breaks are most cytotoxic and if left unrepaired, may jeopardize genetic integrity [24]. It seems that under normal conditions, fractional ATM activities are attenuated by DNA-PK to avoid fast commitment to death and allow faithful DNA repair. The information flows are encoded uniformly possibly potentiating pulse counting in theoretical p53 models [17]. However, DNA-PK inhibitor treatment amplifies and shift the information flows towards the first pulses. The significantly elevated information flows may suppress ‘pulse counting’ while instead lead to rapid cell fate decision. Furthermore, physiologically relevant DDR occurs following very few p53 pulses or even before completion of the first pulse [25], implying the importance of prolonged first pulses under DNA-PK inhibition. Therefore, forced amplification of the first p53 pulses by DNA-PK inhibition might possibly intensify DDR in tumor cells in therapeutics.

To date, rich p53 dynamics other than uniform pulses have been identified [10, 13]. The shift from sustained pulses to monotonic increasing pattern will lead to altered cell fate [10]. Since the dynamics of p53 *per se* can determine cell fate [9], the dynamic change in p53 dynamics with DNA-PK inhibitor has unraveled a hidden layer in p53 mediated cell fate decision [9, 10, 15]. The prolonged p53 pulses have shown a dose dependence (Figure 2, as well as in ref.[15]). The dose dependence using DNA-PK inhibitor is similar to the p53 impulse in response to ultraviolet (UV) light [13]. The implication deserves further investigation.

Our model has several limitations. We used a three-component model to describe the kinetic interplay among PIKK members. However, there exist other processes during DSB repair [24]. Meanwhile, besides NHEJ and homologous recombination (HR), at least two alternative pathways, namely alternative end joining (alt-EJ) and single-strand annealing (SSA) are critically involved [1]. Recently, Buisson et al. identified a concerted role for ATR, DNA-PK and Chk1 during replication stress [26]. In addition, Wip1 also dephosphorylates and inhibits Chk1 [27]. This may create novel negative feedback in p53 signaling. We did not incorporate these effects for simplicity. Our model, however, may highlight some important factors in the novel and complex interplay among PIKK family and p53 signaling. With more sophisticated modeling, deeper mechanistic insights will be provided in future.

## MATERIALS AND METHODS

### Model construction and stochastic DNA damage repair

The p53 oscillator module consists of two principle negative feedback loops and explicit time delays (Figure 1A) [18]. Kinetic interplay among PIKK family members was incorporated based on recent findings [15, 22, 28, 29]. The stochastic double strand breaks repair were modified from a Two Lesion Kinetic model as previously described [30]. The model was formulated using delay ordinary differential equations (ODEs). The model equations are given in Supplementary Table 1. For details, please refer to supplemental materials.

### Mutual information

The mutual information *I* (X, Y) is a measure of uncertainty. It denotes the reduction of uncertainty in Y if the state of random variable X is known. If we name *H* (X) and *H* (Y) as the entropy of random variables X and Y respectively, we have [31]

(1) I(X,Y)=H(Y)−H(Y|X)

We used kernel density estimation (KDE) to approximate the probability density function *f* (*x*). The one- and two-dimensional estimation for probability density function can be introduced into the mutual information, which is a functional of probability densities

(2) $$\hat{I}(X,Y)=\int_{x}\int_{y}{\hat{f}}_{g}(x,y)\log\frac{{\hat{f}}_{g}(x,y)}{\hat{f}(x)\hat{f}(y)}\text{dxdy}$$

For numerical calculation, we simplified the expression and represented the expression by

(3) $$\hat{I}(X,Y)=\frac{1}{N}\sum_{i=1}^{N}\log\frac{{\hat{f}}_{}(x_{i},y_{i})}{\hat{f}(x_{i})\hat{f}(y_{i})}$$

where we sampled *N* times from a multivariate Gaussians with mean (*x_i_, y_i_*) and applied the KDE. Copula transformation was also used to split the data into quantiles [31]. For details regarding calculating mutual information, please refer to Mc Mahon *et al*.’s work [31] and the custom codes are available upon request.

### Local parameter sensitivity

Local parameter sensitivity analysis provides dynamic responses to an infinitesimal disturbance in kinetic parameters. A dynamic system can be defined by ***x****’=F(****x****,* ***p***), where ***x*** and ***p*** donate state vector and parameter vector for the system, respectively. Pulse amplitude and width sensitivity capture the variations of relative amplitude and full-width at half-maximum (FWHM) in response to parametric alterations. The relative amplitude describes the difference between the peak and trough [15]. The FWHM was defined as previously described (see Supplementary Figure 1B in [15]). Briefly, the FWHM describes the difference between the two consecutive time points in the same pulse at which the p53 level is equal to half of its maximum. Relative amplitude sensitivity ***S***_*A*_ and FWHM sensitivity ***S***_*W*_ are defined as

(4) SA=∂AA∂pp=∂ln(A)∂ln(p)

(5) SW=∂WW∂pp=∂ln(W)∂ln(p)

where *A* and *W* denote relative amplitude and FWHM, respectively. Note that these normalized sensitivities are only locally valid in parameter space.

### Stochastic simulation for p53 oscillator

As the maximum level of dynamic species reaches 10^5^, we implemented the stochastic simulation by binomial τ-leap delay method according to Chatterjee et al and Leier et al’s work [32, 33]. The kinetic delays were varied by 10% around the reference values during the simulation. We further assumed that the transcription is burst-like and the burst size positively correlated with the amount of transcription factors [34]. The codes regarding stochastic simulation of our model are available upon request.

### Model simulation

The delay differential equations were numerically integrated using the dde23 solver. Both stochastic and deterministic simulations were performed using MATLAB (MathWork, Version 7.12.0.635, R2011a).

## SUPPLEMENTARY DATA FIGURES AND TABLES





## Abbreviations

ATM: ataxia telangiectasia-mutated
ATR: ataxia telangiectasia and Rad3-related protein
DNA-PK: DNA-dependent protein kinase
FWHM: full-width at half-maximum
KDE: kernel density estimation
PIKK: phosphatidylinositol 3-kinase (PI3K)-like kinase
DSB: double strand break
DDR: DNA damage response
