# Supplementary material for: Modeling amplified p53 responses under DNA-PK inhibition in DNA damage response
Source: Oncotarget. 2017 Feb 3;8(10):17105–14. doi: 10.18632/oncotarget.15062 (PMC5370026; doi:10.18632/oncotarget.15062)
Supplement: Supplementary file 1 [file oncotarget-08-17105-s001.pdf]

Modeling amplified p53 responses under DNA-PK inhibition in DNA damage response

SUPPLEMENTARY DATA

MATERIALS AND METHODS

Model construction

The model consists of 11 species and 65 kinetic parameters. The schematic diagram is shown in Supplementary Figure 1A and the model equations are listed in Supplementary Table 2. For p53, MDM2 and WIP1, we incorporated both mRNA (*italics*) and protein. Total levels of class-IV phosphoinositide 3-kinase (PI3K)-related kinases (PIKK) named Ataxia-telangiectasia-mutated (ATM), ataxia telangiectasia and Rad3-related (ATR) and DNA-dependent protein kinase (DNA-PK) were set to be constant during the simulation [1]. Eq.1 and Eq.2 describe the production and degradation of *p53* and *mdm2* mRNA. The degradation rate of *p53* (k_2) was estimated from [2]. The degradation rate constant for *mdm2* (k_4) was adapted from [3]. Eq.2 further incorporated P53 induced *mdm2* expression. It has been shown that P53 is present at *mdm2* promoter even under unstressed conditions and *mdm2* promoter is not tightly associated with nucleosomes [4, 5]. According to 'anti-repression' model, P53 is constitutively active but repressed by MDM2 [4]. Therefore, free p53 may transactivate a subset of genes such as MDM2. Activated p53 by posttranslational modifications may further transcriptionally upregulate downstream targets such as *mdm2* and *wip1* [6]. Therefore, Eq.3 describes *wip1* turnover. The first two terms in Eq.4 show translation and degradation for P53. The combined P53 degradation rates (basal and MDM2-induced) were adjusted to be consistent with experiments [7]. ATM, ATR and DNA-PKcs can all phosphorylate P53 at Ser15 and activate P53 [4, 6]. For simplicity, DNA-PKcs was abbreviated as DNA-PK throughout the text. Previous reports also showed that WIP1 can dephosphorylate activated ATM, and phosphorylated forms of both P53 and MDM2 [8]. Eq.5 describes the conversion from P53 to activated forms and deactivation by WIP1. We also described MDM2 mediated degradation of activated p53 as previously suggested [9]. Eq. 6 shows *mdm2* translation and MDM2 turnover. ATM could phosphorylate MDM2 at Ser395 and induce MDM2 self-degradation [10, 11]. Therefore, we assumed that MDM2 can perform self-degradation once it is phosphorylated in Eq. 6 and Eq. 7. Eq. 8 simply describes *wip1* translation and WIP1 turnover. The first term in Eq. 9 represents DNA damage induced ATM activation. The second term shows ATM trans-autoactivation. WIP1 mediated ATM dephosphorylation was incorporated as described previously [12]. Eq. 10 also covers DNA damage induced ATR activation. It has been demonstrated that ATR can promote its own phosphorylation and activation via either autophosphorylation [13] or CtIP mediated resection [14]. We here described these effects simply by an autophosphorylation and activation term in Eq. 10. Previous experiments show that DNA-PK may undergo ATM mediated phosphorylation or autophosphorylation at several clusters [15] although recent findings discriminate these effects in further details [16]. Therefore, the second and third terms in Eq. 11 characterize ATM catalyzed DNA-PK phosphorylation and autophosphorylation, respectively for simplicity purpose. Basal deactivation rates are incorporated for PIKKs.

Noticeably, we assumed that DNA double strand breaks (DSBs) can directly activate PI3K-like kinases and subsequently induce p53 activation by phosphorylation (Supplementary Figure 1A and Supplementary Table 1). We also incorporated DNA-PK mediated ATM inhibition (Supplementary Table 1, where the ATM mediated activation was multiplied by a coefficient $\frac{1}{k_{13} \cdot [DNA-PK]^{*} + k_{14}}$) since some reports implied that the ATM kinase activity is inhibited by DNA-PK [17, 18]. Earlier events in DSB repair such as MRE11-RAD50-NBS1 (MRN) and mediator of DNA damage checkpoint protein 1 (MDC1) recruitment, phosphorylation of γH2AX foci, binding of the KU70 and KU80 heterodimers to sites of DNA breaks as well as detailed downstream repair events were not incorporated in our model for simplicity [19]. It has been well documented that ATM relays DNA damage signals through Chk2 while ATR and DNA-PK can coordinate activation of downstream effectors via Chk1 [20, 21]. We did not consider Chk1 and Chk2 in our current model for simplicity owing to their linear signaling transduction properties. Inhibition of DNA-PK catalytic subunit (DNA-PKcs) by specific inhibitors was simplified as DNA-PK inhibition or DNA-PKi. The numbers in Supplementary Figure 1A denote the parameters listed in Supplementary Table 2. Notably, numbers corresponding to basal deactivation rate of activated ATM, ATR and DNA-PK (31, 34 and 38) were not shown in the diagram. We further assumed that the inhibitors can completely block the activity of associated kinases. For treatment with ATM

inhibitor, five parameters (k_{12}, k_{21}, k_{28}, k_{29} and k_{36}) were simultaneously set to be zero. For ATR inhibitor, three parameters (k_{12}, k_{32} and k_{33}) were restricted to be zero. For DNA-PK inhibitors, four parameters k_{17}, k_{35}, k_{36} and k_{37} were taken to be zero. Transcriptional and translational delays are adapted from Ma et al. [9]. The kinetic equations are listed in Supplementary Table 1 while the parameters are provided in Supplementary Table 2.

Stochastic DSB repair

This module is a modification of Ma et al's TLK model. According to Ma et al's work, there exist two repair pathways (i.e. fast and slow) [9]. It has been shown that 1 Gy irradiation can generate 30 DSBs [22]. For stochastic simulations, we assumed that the generated DSBs obey a Poisson distribution with the mean 30*x* where the *x* denotes the radiation dose. Ma et al's TLK model focuses on p53 oscillations under stressed conditions. Ma et al assumed that 70% of the total DSBs are repaired by fast kinetics, while remaining breaks are repaired through slow kinetics. For example, if initial DSB is 100, then 70 DSBs are repaired by fast kinetics while the remaining 30 DSBs are fixed by slow kinetics.

Given relatively low number of repair proteins, the DNA repair process was implemented through stochastic simulation. In DSB repair module, each locus in which a DSB is created can be in one of three states corresponding to intact DSB (state 1), DSB in complex with repair proteins (state 2), and (correctly or incorrectly) fixed DSB (state 3) (Supplementary Figure 1). At time step k, the number of DSBs in corresponding states (i.e. 1, 2 or 3) are represented by D(k), C(k), and F(k), respectively. By using subscripts '1' and '2' to differentiate simple DSBs (fast kinetics) and complex DSBs (slow kinetics), we have $D(k) = D_1(k). + D_2(k)$, $C(k) = C_1(k) + C_2(k)$ and $F(k) = F_1(k) + F_2(k)$. The total number of repair proteins (RP) is assumed to be 20 as previously indicated [9]. At any time, variable repair proteins are free to bind to DSBs. To implement step size control, we chose a relatively small step size ($\Delta t = 0.2$, which is smaller than the shortest τ in τ-leap method in our simulation). That means, even the smallest 'τ' can cover more than one Δt in τ-leap simulation (Supplementary Figure 1). We updated the DNA repair module by consecutive Δt (i.e. n·Δt, where n=τ mod Δt, 'mod' denotes modulus after division). To retain compatibility with τ-leap size, the last step size within the current 'τ' was set to be τ-n·Δt. We simply modified the fixation rate of double strand breaks (k_{fix1}) as: $k_{fix1} = k_{fix1'} \cdot (ATM^* + ATR^* + [DNA\text{-}PK]^*)/3$ as activated ATM, ATR and DNA-PK can participate in the DSB repair process by various ways. The Monte Carlo algorithm for the evolution of DSBs during time [0, *tfinal*] is as follows:

1. Set the initial conditions. The initial total number of DSBs (DSB_T) was generated by a Poisson distribution. Set t=0. We further set $D_1(0) = 0.7 \cdot DSB_T$, $D_2(0) = 0.3 \cdot DSB_T$, $C_1(0) = C_2(0) = 0$ and $F_1(0) = F_2(0) = 0$. Given that initially all repair proteins are free, the simulation starts with total RP=20. There is no DSB until intrinsic DSBs are encountered. If a new DSB occurs, we assume that it is in state 1 (i.e. intact DSB).

2. Increment time. Set t=t+Δt (if not specified). Let k= t/Δt.

3. Update the states for each of the damage sites controlled by fast repair. Compute the transition probabilities as follows:

From state 1 to state 2,
$P_{D1\to C1} = RP[k_{fb1} + k_{cross}(D_1(k-1) + D_2(k-1))]\Delta t$.
From state 2 to state 1,
$P_{C1\to D1} = k_{rb1}\Delta t$.
From state 2 to state 3,
$P_{C1\to F1} = k_{fix1}\Delta t$.

For each DSB, generate a value X from a uniform distribution with 0 and 1. If the DSB is in state 1, a transition to state 2 occurs if $0 \le X < P_{D1\to C1}$, while it stays in state 1 if $P_{D1\to C1} \le X \le 1$. If the damage is in state 2, a transition to state 1 occurs if $0 \le X < P_{C1\to D1}$, or a transition to state 3 occurs if $P_{C1\to D1} \le X < P_{C1\to D1} + P_{C1\to F1}$, or no transition occurs if $P_{C1\to D1} + P_{C1\to F1} \le X \le 1$. If the damage is in state 3, it stays in state 3 (i.e., state 3 is absorbing). Set RP=RP-1 if transition from state 1 to state 2 occurs; set RP = RP + 1 if transition from state 2 to state 1 occurs; otherwise RP remains the same. Count the number of fast repaired breaks at time t in states 1, 2, and 3 to be $D_1(k)$, $C_1(k)$, and $F_1(k)$, respectively.

4. Update the states for each of the damage sites controlled by slow repair. Compute the transition probabilities as follows:

From state 1 to state 2,
$P_{D2\to C2} = RP[k_{fb2} + k_{cross}(D_1(k-1) + D_2(k-1))]\Delta t$.
From state 2 to state 1,
$P_{C2\to D2} = k_{rb2}\Delta t$.
From state 2 to state 3,
$P_{C2\to F2} = k_{fix2}\Delta t$.

For each DSB, generate a value X from a uniform distribution with 0 and 1. If the damage in state 1, a transition to state 2 occurs if $0 \le X < P_{D2\to C2}$, while it stays in state 1 if $P_{D2\to C2} \le X \le 1$. If the damage is in state 2, a transition to state 1 occurs if $0 \le X < P_{C2\to D2}$, or a transition to state 3 occurs if $P_{C2\to D2} \le X < P_{C2\to D2} + P_{C2\to F2}$, or no transition occurs if $P_{C2\to D2} + P_{C2\to F2} \le X \le 1$. If the damage is in state 3, it stays in state 3 (i.e., state 3 is absorbing). Set RP = RP-1 if transition from state 1 to state 2 occurs; set RP = RP + 1 if transition from state 2 to state 1 occurs; otherwise RP remains the same. Count the number of fast repaired breaks at time t in states 1, 2, and 3 to be $D_2(k)$, $C_2(k)$, and $F_2(k)$, respectively.

5. Let $D(k) = D_1(k) + D_2(k)$, $C(k) = C_1(k) + C_2(k)$, and $F(k) = F_1(k) + F_2(k)$.

6. Repeat steps 2-5 until t=*tfinal*.

Summarizing the binomial τ-leap stochastic simulation

For a system with N molecular species, the vector $X=(X_1(t),\ldots, X_N(t))$ denotes the number of molecules X_i at time t, $1 \le i \le N$ ($N = 11$ in our model). Each species is involved in M chemical reaction channels ($M = 36$ in our model) represented by R_j, $j=1, 2, \ldots, M$ with a propensity function a_j. Then, $a_j dt$ denotes the probability that one reaction R_j will happen in the time interval $[t, t + dt]$ and the v_{ij} is the stoichiometric coefficient of species S_i in reaction R_j.

In binomial τ-leap method, each of the M reactions is allowed to fire in a given order in $[t, t + \tau]$. The maximum firing number $k_{max}^{(j)}$ is determined by the limiting substrates, i.e. the species which would be completely depleted if the reaction went to completion. Identification of the limiting reactants is the critical step for implementation of the algorithm.

During the stochastic simulations, the parameters should be rescaled by a factor Ω. For kinetic parameters describing zero-order reaction (e.g. with unit μM· min^{-1}), corresponding parameters in stochastic simulations should be multiplied by Ω. For second order reaction parameters (e.g. with unit μM^{-1}· min^{-1}), the corresponding parameters should be divided by Ω. The first order parameters are unchanged (See Supplementary Table 2 for parameters). All reactants are multiplied by Ω. $\Omega = 6\times10^4$ in the model.

Briefly, the binomial τ-leap algorithm is represented as follows:

(1) Obtaining the stoichiometric coefficients v_{ij}, the initial number of all species X(0) and the rate constants.

(2) Setting the initial time, t_0.

(3) Repeat step 4-6 until the final time t_{final} has been reached.

(4) Obtaining the propensity function $a_j(X(t))$ using the current population $X(t)$.

(5) Compute τ and update $t = t + \tau$. τ is defined as follows:

$$\tau = f / \sum_{j=1}^{M} a_j$$

Where f denotes a coarse-grained factor and is set to be 10^4 as suggested [23].

(6) For $j = 1$ to M reactions,

(a) Computing $k_{max}^{(j)} = \min(v_{ij}<0)(\mathrm{int}(X_i'/|v_{ij}|))$, where X' denotes the updated X before execution of any reaction step and int( ) is the largest number of integer function.

(b) Compute $p = a_j\,\tau / k_{max}^{(j)}$, sample k_j from the binomial distribution as defined below:

$$P_{BD}(k_j; p, k_{\max}^{(j)}) = \frac{k_{\max}^{(j)}!}{k_j!(k_{\max}^{(j)} - k_j)!} p^{k_j} (1-p)^{k_{\max}^{(j)}-k_j}$$

BD denotes binomial distribution. If $a_j\,\tau > k_{max}^{(j)}$, we set $p = 1$.

(c) Set $X_i' = X_i' + v_{ij}\,k_j$ for $i = 1, \ldots, N$ if $v_{ij} < 0$.

(7) Go to step 6a.

(8) Update $X_i = X_i + v_{ij}\,k_j$, for $i = 1, \ldots, N$.

(9) Go to step 3.

The reactions in stochastic simulations were listed in Supplementary Table 3.

The stoichiometric matrix $S=[v_{ij}]$ is provided below:

[1 0 0 0 0 0 0 0 0 0 0
-1 0 0 0 0 0 0 0 0 0 0
0 1 0 0 0 0 0 0 0 0 0
0 -1 0 0 0 0 0 0 0 0 0
0 1 0 0 0 0 0 0 0 0 0
0 0 1 0 0 0 0 0 0 0 0
0 0 -1 0 0 0 0 0 0 0 0
0 0 1 0 0 0 0 0 0 0 0
0 0 0 1 0 0 0 0 0 0 0
0 0 0 -1 0 0 0 0 0 0 0
0 0 0 -1 0 0 0 0 0 0 0
0 0 0 -1 1 0 0 0 0 0 0
0 0 0 1 -1 0 0 0 0 0 0
0 0 0 0 -1 0 0 0 0 0 0
0 0 0 0 0 1 0 0 0 0 0
0 0 0 0 0 -1 0 0 0 0 0
0 0 0 0 0 -1 1 0 0 0 0
0 0 0 0 0 1 -1 0 0 0 0
0 0 0 0 0 0 -1 0 0 0 0
0 0 0 0 0 0 0 1 0 0 0
0 0 0 0 0 0 0 -1 0 0 0
0 0 0 0 0 0 0 0 1 0 0
0 0 0 0 0 0 0 0 -1 0 0
0 0 0 0 0 0 0 0 -1 0 0
0 0 0 0 0 0 0 0 1 0 0
0 0 0 -1 1 0 0 0 0 0 0
0 0 0 -1 1 0 0 0 0 0 0
0 0 0 0 0 -1 0 0 0 0 0
0 0 0 0 0 0 -1 0 0 0 0
0 0 0 0 0 0 0 0 0 1 0
0 0 0 0 0 0 0 0 0 1 0
0 0 0 0 0 0 0 0 0 -1 0
0 0 0 0 0 0 0 0 0 0 1
0 0 0 0 0 0 0 0 0 0 1
0 0 0 0 0 0 0 0 0 0 1
0 0 0 0 0 0 0 0 0 0 -1]

For detailed algorithm, please refer to refs [23, 24].

REFERENCES

1. Hirai, Y., Hayashi, T., Kubo, Y., Hoki, Y., Arita, I., Tatsumi, K., Seyama, T. (2001). X-irradiation induces up-regulation of ATM gene expression in wild-type lymphoblastoid cell lines, but not in their heterozygous or homozygous ataxia-telangiectasia counterparts. Japanese Journal of Cancer Research 92, 710-717.
2. Kim, H., You, S., Foster, L.K., Farris, J., Foster, D.N. (2001). The rapid destabilization of p53 mRNA in immortal chicken embryo fibroblast cells. Oncogene 20, 5118-23.
3. Hsing, A., Faller, D.V., Vaziri, C. (2000). DNA-damaging aryl hydrocarbons induce Mdm2 expression via p53-independent post-transcriptional mechanisms. J Biol Chem 275, 26024-31.
4. Kruse, J.P., Gu, W. (2009). Modes of p53 regulation. Cell 137, 609-22.
5. Vousden, K.H., Prives, C. (2009). Blinded by the Light: The Growing Complexity of p53. Cell 137, 413-31.
6. Meek, D.W. (2015). Regulation of the p53 response and its relationship to cancer. Biochem J 469, 325-46.
7. Ju, J., Schmitz, J.C., Song, B., Kudo, K., Chu, E. (2007). Regulation of p53 expression in response to 5-fluorouracil in human cancer RKO cells. Clinical Cancer Research 13, 4245-51.
8. Shimada, M., Nakanishi, M. (2013). Response to DNA damage: why do we need to focus on protein phosphatases? Front Oncol 3, 8.
9. Ma, L., Wagner, J., Rice, J.J., Hu, W., Levine, A.J., Stolovitzky, G.A. (2005). A plausible model for the digital response of p53 to DNA damage. Proc Natl Acad Sci U S A 102, 14266-71.
10. Maya, R. et al. (2001). ATM-dependent phosphorylation of Mdm2 on serine 395: role in p53 activation by DNA damage. Genes Dev 15, 1067-77.
11. Stommel, J.M., Wahl, G.M. (2004). Accelerated MDM2 auto-degradation induced by DNA-damage kinases is required for p53 activation. EMBO J 23, 1547-56.
12. Batchelor, E., Mock, C.S., Bhan, I., Loewer, A., Lahav, G. (2008). Recurrent initiation: a mechanism for triggering p53 pulses in response to DNA damage. Mol Cell 30, 277-89.
13. Liu, S. et al. (2011). ATR autophosphorylation as a molecular switch for checkpoint activation. Mol Cell 43, 192-202.
14. Peterson, S.E., Li, Y., Wu-Baer, F., Chait, B.T., Baer, R., Yan, H., Gottesman, M.E., Gautier, J. (2013). Activation of DSB processing requires phosphorylation of CtIP by ATR. Mol Cell 49, 657-67.
15. Davis, A.J., Chen, B.P., Chen, D.J. (2014). DNA-PK: a dynamic enzyme in a versatile DSB repair pathway. DNA Repair (Amst) 17, 21-9.
16. Jiang, W. et al. (2015). Differential phosphorylation of DNA-PKcs regulates the interplay between end-processing and end-ligation during nonhomologous end-joining. Mol Cell 58, 172-85.
17. Hollingworth, R., Skalka, G.L., Stewart, G.S., Hislop, A.D., Blackbourn, D.J., Grand, R.J. (2015). Activation of DNA Damage Response Pathways during Lytic Replication of KSHV. Viruses 7, 2908-27.
18. Finzel, A., Grybowski, A., Strasen, J., Cristiano, E., Loewer, A. (2016). Hyperactivation of ATM upon DNA-PKcs inhibition modulates p53 dynamics and cell fate in response to DNA damage. Molecular Biology of the Cell 27, 2360-7.
19. Morgan, M.A., Lawrence, T.S. (2015). Molecular Pathways: Overcoming Radiation Resistance by Targeting DNA Damage Response Pathways. Clinical Cancer Research 21, 2898-904.
20. Buisson, R., Boisvert, J.L., Benes, C.H., Zou, L. (2015). Distinct but Concerted Roles of ATR, DNA-PK, and Chk1 in Countering Replication Stress during S Phase. Mol Cell 59, 1011-24.
21. Awasthi, P., Foiani, M., Kumar, A. (2016). ATM and ATR signaling at a glance. J Cell Sci 129, 1285.
22. Vilenchik, M.M., Knudson, A.G. (2003). Endogenous DNA double-strand breaks: Production, fidelity of repair, and induction of cancer. Proceedings of the National Academy of Sciences of the United States of America 100, 12871-12876.
23. Chatterjee, A., Vlachos, D.G., Katsoulakis, M.A. (2005). Binomial distribution based tau-leap accelerated stochastic simulation. J Chem Phys 122, 024112.
24. Leier, A., Marquez-Lago, T.T., Burrage, K. (2008). Generalized binomial tau-leap method for biochemical kinetics incorporating both delay and intrinsic noise. Journal of Chemical Physics 128.

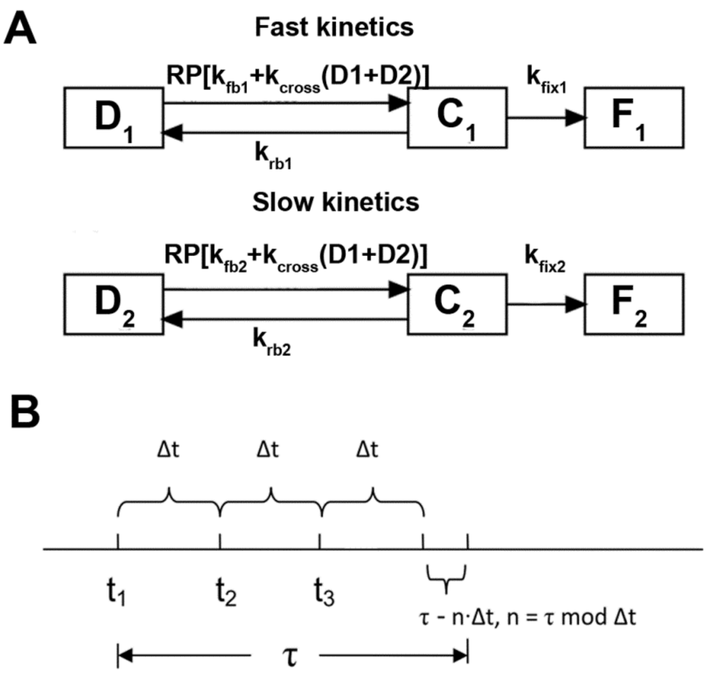

Supplementary Figure 1: Representation of the DSB module. A. Two Lesion Kinetic (TLK) model for DSB repair. Each locus can be in one of three states corresponding to intact DSB (state 1), DSB in complex with repair proteins (state 2), and fixed DSB (state 3). At time step k, the number of DSBs in corresponding states (i.e. 1, 2 or 3) are represented by D(k), C(k), and F(k), respectively. Subscripts '1' and '2' differentiate simple DSBs (fast kinetics) and complex DSBs (slow kinetics), respectively. **B.** Compatibility between Monte Carlo time steps and τ-Leap size. Δt represents Monte Carlo step size while τ denotes τ-Leap size. The smallest leap size τ is larger than Δt ($\Delta t = 0.2$).

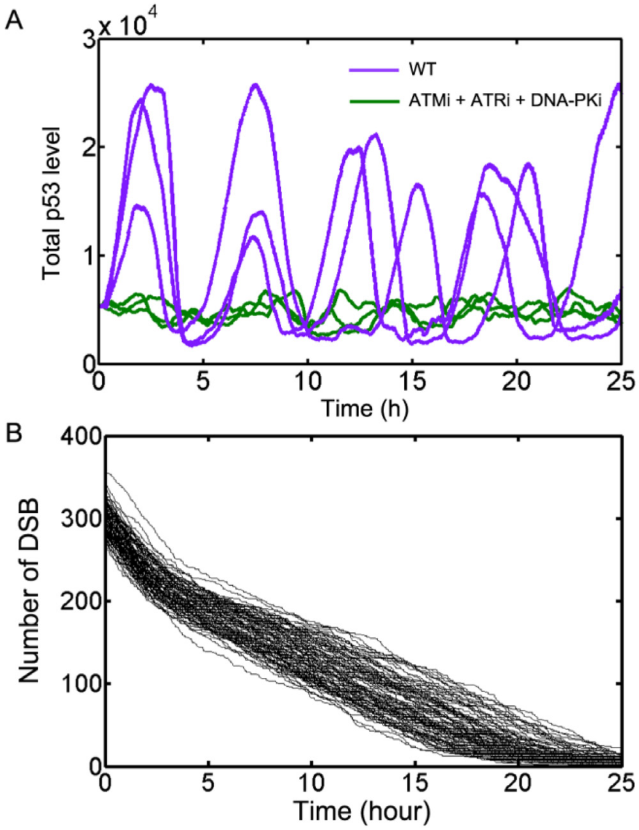

Supplementary Figure 2: Temporal dynamics of p53 and DSB. A. *In silico* stochastic simulation of p53 dynamics in cells either untreated (violet) or treatment with all PIKK inhibitors (green). IR = 10 Gy. Three stochastic simulation runs are shown for each condition. **B.** Temporal evolution of DSB repair under 10 Gy irradiation. 100 representative plots were shown.

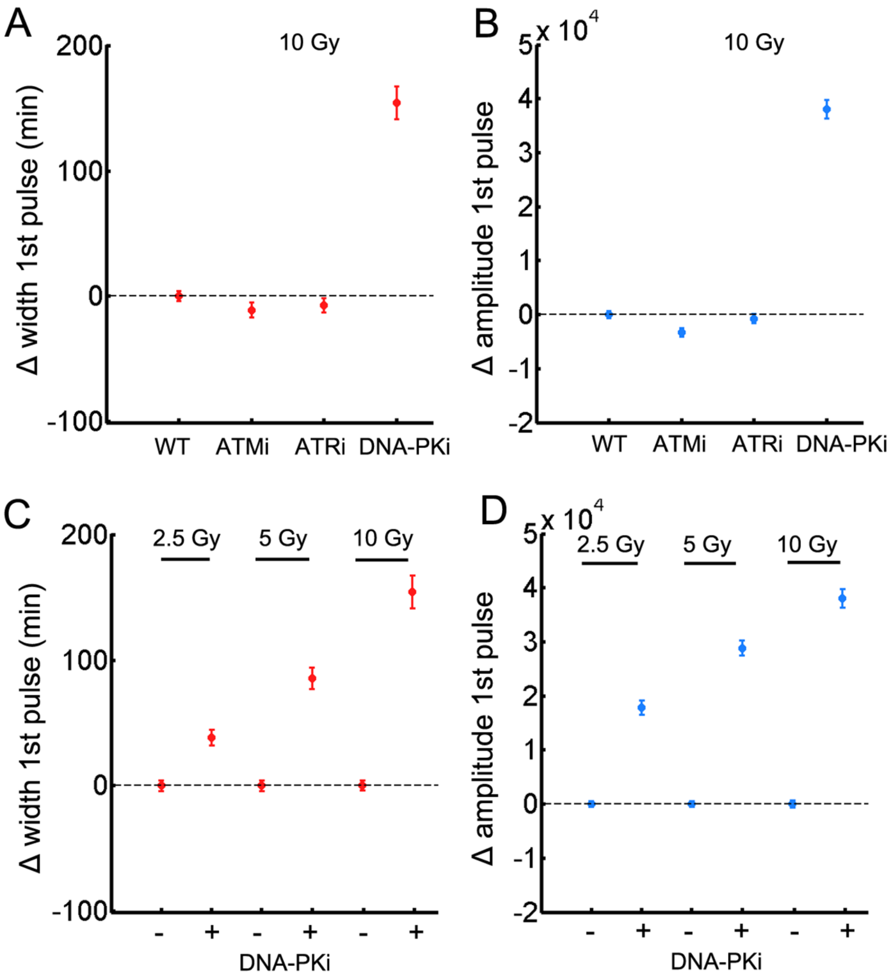

Supplementary Figure 3: Statistical results of Supplementary Figure 2. The estimated changes of the mean pulse width (**A.** red) and amplitude (**B.** blue) for the first p53 pulses under indicated conditions. Quantification of the changes in pulse width **C.** and amplitude **D.** in cells untreated or treated with DNA-PK inhibitors under 2.5 Gy, 5 Gy and 10 Gy conditions. Error bars represent 95% confidence intervals.

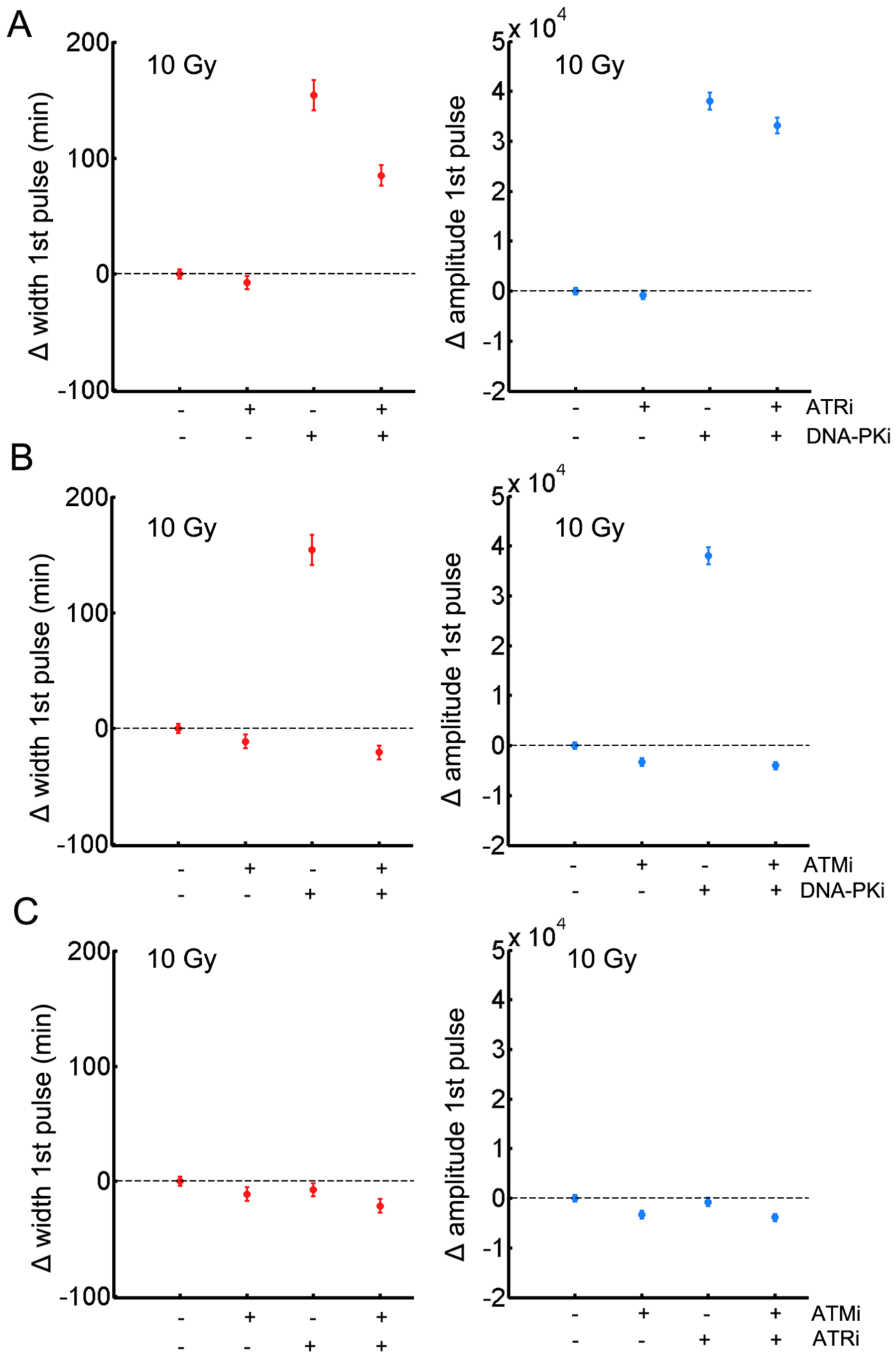

Supplementary Figure 4: Statistical results of Figure 3. The estimated changes of the mean FWHM (**A.** red) and amplitude (**B.** blue) for the first p53 pulses under the circumstances when the PIKK inhibitors were used in combination or alone. The error bars denote 95% confidence intervals. The groups with PIKK inhibitors were compared with untreated groups.

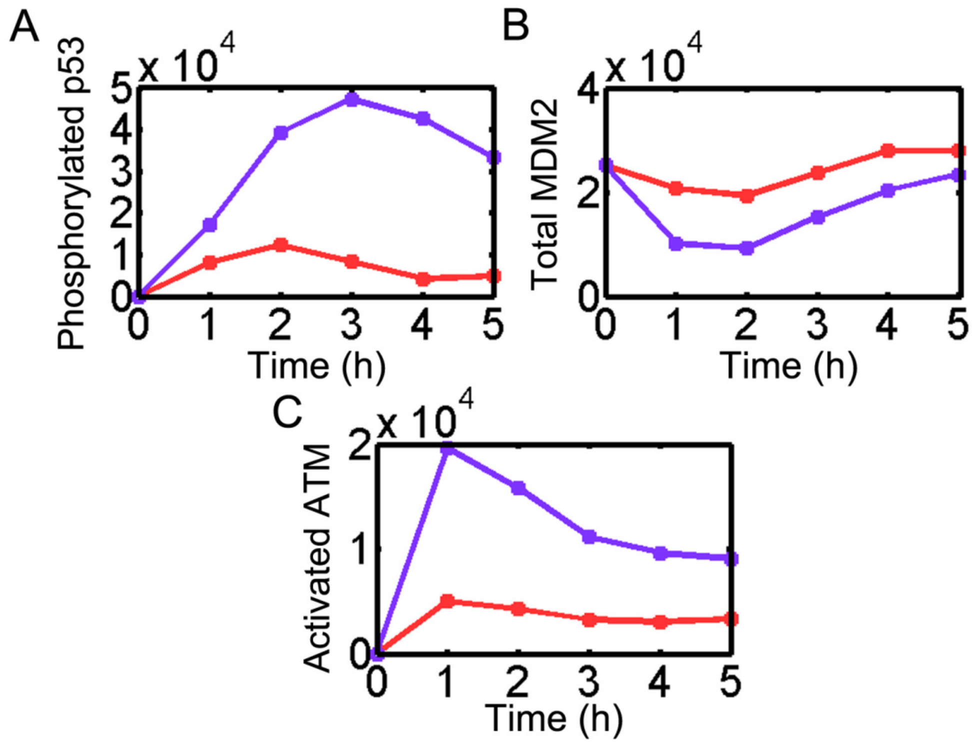

Supplementary Figure 5: Simulation of p53, MDM2 and activated ATM with or without DNA-PK inhibition. Temporal dynamics of total p53 **A.** MDM2 **B.** and activated ATM **C.** within first 5 hours post irradiation as determined in Finzel et al.' work [18] with (blue) or without (red) DNA-PK inhibitor treatment. Data were averaged from 1000 simulations.

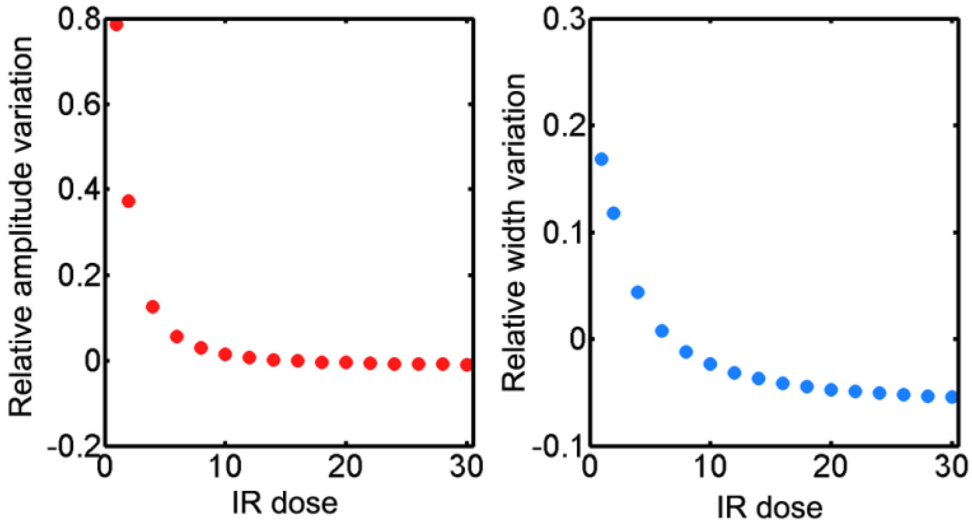

Supplementary Figure 6: Relative changes of pulse amplitude and width under DNA-PK inhibitor treatment and wild type conditions. The difference for pulse amplitude (left) and period (right) between DNA-PKi and WT conditions were evaluated and normalized to the values under WT conditions.

Supplementary Table 1: Dynamic equations for the model

$$\frac{dp53}{dt} = k_1 - k_2 \cdot p53 \qquad \text{Eq.1}$$

$$\frac{dmdm2}{dt} = k_3 - k_4 \cdot mdm2 + k_5 \cdot (P53(t-\tau_1) + P53p(t-\tau_1))^4 / [(P53(t-\tau_1) + P53p(t-\tau_1))^4 + K_1^4] \qquad \text{Eq.2}$$

$$\frac{dwip1}{dt} = k_6 - k_7 \cdot wip1 + k_8 \cdot P53p(t-\tau_2)^4 / [P53p(t-\tau_2)^4 + K_2^4] \qquad \text{Eq.3}$$

$$\begin{aligned}\frac{dP53}{dt} = {} & k_9 \cdot p53 - k_{10} \cdot P53 - k_{11} \cdot MDM2 \cdot P53 / (P53 + K_3) - k_{12}^{'} \cdot ATM^* \cdot P53 / (P53 + K_4) \\ & + k_{15} \cdot WIP1 \cdot P53p / (P53p + K_5) - k_{16} \cdot ATR^* \cdot P53 / (P53 + K_6) \\ & - k_{17} \cdot [DNA-PK]^* \cdot P53 / (P53 + K_7)\end{aligned} \qquad \text{Eq.4}$$

$$\begin{aligned}\frac{dP53p}{dt} = {} & k_{12}^{'} \cdot ATM^* \cdot P53 / (P53 + K_4) - k_{15} \cdot WIP1 \cdot P53p / (P53p + K_5) \\ & + k_{16} \cdot ATR^* \cdot P53 / (P53 + K_6) + k_{17} \cdot [DNA-PK]^* \cdot P53 / (P53 + K_7) - k_{18} \cdot MDM2 \cdot P53p / (P53p + K_8)\end{aligned} \qquad \text{Eq.5}$$

$$\begin{aligned}\frac{dMDM2}{dt} = {} & k_{19} \cdot mdm2(t-\tau_3) - k_{20} \cdot MDM2 - k_{21}^{'} \cdot ATM^* \cdot MDM2 / (MDM2 + K_9) \\ & + k_{22} \cdot WIP1 \cdot MDM2p / (MDM2p + K_{10}) - k_{23} \cdot MDM2p \cdot MDM2 / (MDM2 + K_{11})\end{aligned} \qquad \text{Eq.6}$$

$$\begin{aligned}\frac{dMDM2p}{dt} = {} & -k_{24} \cdot MDM2p + k_{21}^{'} \cdot ATM^* \cdot MDM2 / (MDM2 + K_9) \\ & - k_{22} \cdot WIP1 \cdot MDM2p / (MDM2p + K_{10}) - k_{25} \cdot MDM2p \cdot MDM2p / (MDM2p + K_{12})\end{aligned} \qquad \text{Eq.7}$$

$$\frac{dWIP1}{dt} = k_{26} \cdot wip1(t-\tau_4) - k_{27} \cdot WIP1 \qquad \text{Eq.8}$$

$$\begin{aligned}\frac{dATM^*}{dt} = {} & k_{28}^{'} \cdot DSB \cdot ATM + k_{29}^{'} \cdot ATM^* \cdot ATM / (ATM + K_{13}) \\ & - k_{30} \cdot WIP1 \cdot ATM^* / (ATM^* + K_{14}) - k_{31} \cdot ATM^*\end{aligned} \qquad \text{Eq.9}$$

$$\frac{dATR^*}{dt} = k_{32} \cdot DSB \cdot ATR + k_{33} \cdot ATR^* \cdot ATR / (ATR + K_{15}) - k_{34} \cdot ATR^* \qquad \text{Eq.10}$$

$$\begin{aligned}\frac{d[DNA-PK]^*}{dt} = {} & k_{35} \cdot DSB \cdot [DNA-PK] + k_{36}^{'} \cdot ATM^* \cdot [DNA-PK] / ([DNA-PK] + K_{16}) \\ & + k_{37} \cdot [DNA-PK]^* \cdot [DNA-PK] / ([DNA-PK] + K_{17}) - k_{38} \cdot [DNA-PK]^*\end{aligned} \qquad \text{Eq.11}$$

$$ATM + ATM^* = 1, \quad ATR + ATR^* = 1, \quad [DNA-PK] + [DNA-PK]^* = 1$$

$$DSB = \frac{IR \cdot 30}{IR \cdot 30 + KmDSB}, \quad k_i^{'} = \frac{1}{k_{13} \cdot [DNA-PK]^* + k_{14}} \cdot k_i (i = 12, 21, 28, 29, 36)$$

The equations are formulated with ordinary differential equations and delay differential equations. The italics denote mRNA species.

Supplementary Table 2: Model parameters and description

See Supplementary File 1

Supplementary Table 3: The reaction number in stochastic simulation

No.	Reactions
1	k_1
2	$k_2 \cdot p53$
3	k_3
4	$k_4 \cdot mdm2$
5	$k_5 \cdot (\mathrm{P53p+P53})^4/[(\mathrm{P53p+P53})^4 + K_1^4]$
6	k_6
7	$k_7 \cdot wip1$
8	$k_8 \cdot \mathrm{P53p}^4/(\mathrm{P53p}^4 + K_2^4)$
9	$k_9 \cdot p53$
10	$k_{10} \cdot \mathrm{P53}$
11	$k_{11} \cdot \mathrm{P53} \cdot \mathrm{MDM2}/(\mathrm{P53} + K_3)$
12	$k_{12} \cdot \mathrm{P53} \cdot \mathrm{ATM}^*/(k_{13} \cdot \mathrm{DNA\text{-}PK}^* + k_{14})/(\mathrm{P53} + K_4)$
13	$k_{15} \cdot \mathrm{P53p} \cdot \mathrm{WIP1}/(\mathrm{P53p} + K_5)$
14	$k_{18} \cdot \mathrm{P53p} \cdot \mathrm{MDM2}/(\mathrm{P53p} + K_8)$
15	$k_{19} \cdot mdm2$
16	$k_{20} \cdot \mathrm{MDM2}$
17	$k_{21} \cdot \mathrm{ATM}^* \cdot \mathrm{MDM2}/(k_{13} \cdot \mathrm{DNA\text{-}PK}^* + k_{14})/(\mathrm{MDM2} + K_9)$
18	$k_{22} \cdot \mathrm{WIP1} \cdot \mathrm{MDM2p}/(\mathrm{MDM2p} + K_{10})$
19	$k_{24} \cdot \mathrm{MDM2p}$
20	$k_{26} \cdot wip1$
21	$k_{27} \cdot \mathrm{WIP1}$
22	$k_{28}/(k_{13} \cdot \mathrm{DNA\text{-}PK}^* + k_{14}) \cdot \mathrm{DSB} \cdot (1-\mathrm{ATM}^*)$
23	$k_{30} \cdot \mathrm{WIP1} \cdot \mathrm{ATM}^*/(\cdot \mathrm{ATM}^* + K_{14})$
24	$k_{31} \cdot \mathrm{ATM}^*$
25	$k_{29}/(k_{13} \cdot \mathrm{DNA\text{-}PK}^* + k_{14}) \cdot \mathrm{ATM}^* \cdot (1-\mathrm{ATM}^*)/((1-\mathrm{ATM}^*) + K_{13})$
26	$k_{16} \cdot \mathrm{ATR}^* \cdot \mathrm{P53}/(\mathrm{P53} + K_6)$
27	$k_{17} \cdot \mathrm{P53}/(\mathrm{P53} + K_7)$
28	$k_{23} \cdot \mathrm{MDM2p} \cdot \mathrm{MDM2}/(\mathrm{MDM2} + K_{11})$
29	$k_{25} \cdot \mathrm{MDM2p} \cdot \mathrm{MDM2p}/(\mathrm{MDM2p} + K_{12})$
30	$k_{32} \cdot \mathrm{DSB} \cdot (1-\mathrm{ATR}^*)$
31	$k_{33} \cdot \mathrm{ATR}^* \cdot (1-\mathrm{ATR}^*)/(1-\mathrm{ATR}^* + K_{15})$
32	$k_{34} \cdot \mathrm{ATR}^*$
33	$k_{35} \cdot \mathrm{DSB} \cdot (1-\mathrm{DNA\text{-}PK}^*)$
34	$k_{36}/(k_{13} \cdot \mathrm{DNA\text{-}PK}^* + k_{14}) \cdot \mathrm{ATM}^* \cdot (1-\mathrm{DNA\text{-}PK}^*)/(1-\mathrm{DNA\text{-}PK}^* + K_{16})$
35	$k_{37} \cdot \mathrm{DNA\text{-}PK}^* \cdot (1-\mathrm{DNA\text{-}PK}^*)/(1-\mathrm{DNA\text{-}PK}^* + K_{17})$
36	$k_{38} \cdot \mathrm{DNA\text{-}PK}^*$
