# Supplementary material for: Modeling amplified p53 responses under DNA-PK inhibition in DNA damage response
Source: Oncotarget. 2017 Feb 3;8(10):17105–14. doi: 10.18632/oncotarget.15062 (PMC5370026; doi:10.18632/oncotarget.15062)
Supplement: Supplementary file 2 [file oncotarget-08-17105-s002.docx]

**Table S2:** Model parameters and description

| **Parameters** | **Description** | **Value ^a^** |
| --- | --- | --- |
| [*p53*]_0_ | Initial concentration P53 mRNA | 0.1000 |
| [*mdm2*]_0_ | Initial concentration of MDM2 mRNA | 0.0977 |
| [*wip1*]_0_ | Initial concentration of WIP1 mRNA | 0.0400 |
| [P53]_0_ | Initial concentration of P53 | 0.0886 |
| [MDM2]_0_ | Initial concentration of MDM2 | 0.4188 |
| [WIP1]_0_ | Initial concentration of WIP1 | 0.2667 |
| [ATM]_0_ | Unphosphorylated ATM | 1 |
| [ATR]_0_ | Unphosphorylated ATR | 1 |
| [DNA-PK]_0_ | Unphosphorylated DNA-PK | 1 |
| *k_1_* | Production rate of *p53* | 0.0005 |
| *k_2_* | Degradation rate of *p53* | 0.005 |
| *k_3_* | Production rate of *mdm2* | 0.001 |
| *k_4_* | Degradation rate of *mdm2* | 0.02 |
| *k_5_* | P53 dependent *mdm2* transcription rate | 0.0025 |
| *k_6_* | Production rate of *wip1* | 0.002 |
| *k_7_* | Degradation rate of *wip1* | 0.05 |
| *k_8_* | P53 dependent *wip1* transcription rate | 0.0025 |
| *k_9_* | Translation rate of P53 | 0.1 |
| *k_10_* | Degradation rate of P53 | 0.005 |
| *k_11_* | MDM2 dependent P53 degradation | 0.1 |
| *k_12_* | ATM* induced P53 phosphorylation | 1 |
| *k_13_* | [DNA-PK] mediated ATM^*^ inhibition | 2 |
| *k_14_* | Equilibrium constant for ATM^*^ inhibition | 1 |
| *k_15_* | WIP1 induced P53p dephosphorylation | 0.05 |
| *k_16_* | ATR* induced P53 phosphorylation | 0.5 |
| *k_17_* | [DNA-PK]* induced P53 phosphorylation | 0.5 |
| *k_18_* | MDM2 dependent P53p degradation | 0.025 |
| *k_19_* | *mdm2* translation rate | 0.15 |
| *k_20_* | Basal MDM2 degradation rate | 0.035 |
| *k_21_* | ATM^*^ induced MDM2 phosphorylation | 0.1 |
| *k_22_* | WIP1 induced MDM2p dephosphorylation | 0.1 |
| *k_23_* | MDM2p induced MDM2 degradation | 0.1 |
| *k_24_* | Basal MDM2p degradation rate | 0.14 |
| *k_25_* | MDM2p induced self-degradation | 0.1 |
| *k_26_* | *wip1* translation rate | 0.1 |
| *k_27_* | Basal WIP1 degradation rate | 0.015 |
| *k_28_* | DSB induced ATM^*^ activation | 0.1 |
| *k_29_* | ATM^*^ autoactivation | 0.1 |
| *k_30_* | WIP1 mediated ATM^*^ dephosphorylation rate | 0.3 |
| *k_31_* | basal ATM^*^ dephosphorylation rate | 0.1 |
| *k_32_* | DSB induced ATR^*^ activation | 0.1 |
| *k_33_* | ATR^*^ autoactivation | 0.1 |
| *k_34_* | basal ATR^*^ dephosphorylation rate | 0.1 |
| *k_35_* | DSB induced [DNA-PK]^*^ activation | 0.1 |
| *k_36_* | ATM^*^ induced [DNA-PK]^*^ activation rate | 0.1 |
| *k_37_* | [DNA-PK]^*^ autoactivation | 0.1 |
| *k_38_* | basal [DNA-PK]^*^ dephosphorylation rate | 0.1 |
| *K_mDSB_* | Equilibrium constant for kinase activation | 100 |
| *K_1_* | *t.c.* P53 induced *mdm2* expression | 0.1 |
| *K_2_* | *t.c.* P53 induced *wip1* expression | 0.1 |
| *K_3_* | *m.c.* MDM2 induced P53 degradation | 0.3 |
| *K_4_* | *m.c.* ATM^*^ induced P53 phosphorylation | 0.1 |
| *K_5_* | *m.c.* WIP1 induced P53p dephosphorylation | 0.2 |
| *K_6_* | *m.c.* ATR^*^ induced P53 phosphorylation | 0.1 |
| *K_7_* | *m.c.* [DNA-PK]^*^ induced P53 phosphorylation | 0.1 |
| *K_8_* | *m.c.* MDM2 induced P53p degradation | 0.03 |
| *K_9_* | *m.c.* ATM^*^ induced MDM2 phosphorylation | 0.1 |
| *K_10_* | *m.c.* WIP1 induced MDM2p dephosphorylation | 0.2 |
| *K_11_* | *m.c.* MDM2p induced MDM2 degradation | 0.3 |
| *K_12_* | *m.c.* MDM2p induced MDM2p degradation | 0.3 |
| *K_13_* | *m.c.* ATM^*^ autophosphorylation | 0.1 |
| *K_14_* | *m.c.* WIP1 induced ATM^*^ dephosphorylation | 0.3 |
| *K_15_* | *m.c.* ATR^*^ autophosphorylation | 0.1 |
| *K_16_* | *m.c.* ATM^*^ induced DNA-PK phosphorylation | 0.1 |
| *K_17_* | *m.c.* [DNA-PK]^*^ autophosphorylation | 0.1 |
| *τ_1_* | transcriptional delay for *mdm2* | 30 |
| *τ_2_* | transcriptional delay for *wip1* | 30 |
| *τ_3_* | translation delay for *mdm2* | 10 |
| *τ_4_* | translation delay for *wip1* | 10 |
| *k_fb1_* | association rate of fast kinetics | 0.05 |
| *k_rb1_* | dissociation rate of fast kinetics | 0.01 |
| *k_fix1’_* | repair rates of fast kinetics | 0.05 |
| *f_scale_* | scale parameter | 7 |
| *k_cross_* | cross fixation rate | 0.002 |

**a:** The first and second order rate constants are expressed in units of min^-1^ and μM^-1^∙ min^-1^, respectively. The production rates are expressed in units of μM∙ min^-1^. The initial conditions were set to be zero unless specified. All delays take the unit min. The parameters in DSB repair module are in units of 1. *t.c.* denotes threshold concentration, *m.c.* represents Michaelis constant. The remaining parameters *k_fb2_, k_fix2_*, and *k_rb2_* can be obtained through dividing by the scaling parameter. We modified the parameter *k_fix1_ as:* *k_fix1_*= *k_fix1’_* ∙ (*ATM^*^+ATR^*^+[DNA-PK]^*^*)/3.
